# Genetic causes of primary aldosteronism

**DOI:** 10.1038/s12276-019-0337-9

**Published:** 2019-11-06

**Authors:** Eric Seidel, Julia Schewe, Ute I. Scholl

**Affiliations:** 1 Charité—Universitätsmedizin Berlin, Corporate Member of Freie Universität Berlin, Humboldt-Universität zu Berlin, and Berlin Institute of Health, Department of Nephrology and Medical Intensive Care, 13353 Berlin, Germany; 2 Berlin Institute of Health (BIH), 10178 Berlin, Germany; 3 Charité—Universitätsmedizin Berlin, BCRT—BIH Center for Regenerative Therapies, 10178 Berlin, Germany

**Keywords:** Adrenal gland diseases, Hypertension

## Abstract

Primary aldosteronism is characterized by at least partially autonomous production of the adrenal steroid hormone aldosterone and is the most common cause of secondary hypertension. The most frequent subforms are idiopathic hyperaldosteronism and aldosterone-producing adenoma. Rare causes include unilateral hyperplasia, adrenocortical carcinoma and Mendelian forms (familial hyperaldosteronism). Studies conducted in the last eight years have identified somatic driver mutations in a substantial portion of aldosterone-producing adenomas, including the genes *KCNJ5* (encoding inwardly rectifying potassium channel GIRK4), *CACNA1D* (encoding a subunit of L-type voltage-gated calcium channel Ca_V_1.3), *ATP1A1* (encoding a subunit of Na^+^/K^+^-ATPase), *ATP2B3* (encoding a Ca^2+^-ATPase), and *CTNNB1* (encoding ß-catenin). In addition, aldosterone-producing cells were recently reported to form small clusters (aldosterone-producing cell clusters) beneath the adrenal capsule. Such clusters accumulate with age and appear to be more frequent in individuals with idiopathic hyperaldosteronism. The fact that they are associated with somatic mutations implicated in aldosterone-producing adenomas also suggests a precursor function for adenomas. Rare germline variants of *CYP11B2* (encoding aldosterone synthase), *CLCN2* (encoding voltage-gated chloride channel ClC-2), *KCNJ5*, *CACNA1H* (encoding a subunit of T-type voltage-gated calcium channel Ca_V_3.2), and *CACNA1D* have been reported in different subtypes of familial hyperaldosteronism. Collectively, these studies suggest that primary aldosteronism is largely due to genetic mutations in single genes, with potential implications for diagnosis and therapy.

## Hypertension: Understanding the hormone aldosterone

Recent genetic studies are providing a new perspective on a leading cause of high blood pressure, primary aldosteronism (PA). In PA, the hormone aldosterone is overproduced, causing salt retention and leading to high blood pressure, which carries an increased risk of stroke and heart attack. PA can have a variety of causes, making diagnosis tricky; unfortunately, the best method of diagnosis is both invasive and difficult to perform. Ute Scholl at the Berlin Institute of Health in Germany and co-workers have reviewed recent research into PA, revealing that mutations in only a few genes, most of which are involved in calcium regulation, underly most cases. They traced different subforms of PA to different mutations in the same gene. Understanding the underlying genetics will pave the way to better diagnostic and therapeutic tools for PA.

## Introduction

The adrenal salt-retaining steroid hormone aldosterone is produced in the zona glomerulosa (ZG), the outermost zone of the adrenal cortex, from its precursor, cholesterol. In volume depletion, a major stimulus of aldosterone production, angiotensinogen is cleaved by the aspartyl protease renin, leading to the production of angiotensin I, which is then further processed to angiotensin II (ATII) by angiotensin converting enzyme (ACE). The second major stimulus of aldosterone production is hyperkalemia, which directly raises adrenal aldosterone production^1^. Aldosterone, by binding to mineralocorticoid receptors in the distal convoluted tubule, the connecting tubule and the cortical collecting duct of the kidney, increases the abundance or activity of numerous proteins, including the Na^+^/K^+^-ATPase and the epithelial sodium channel^2^. Consequently, aldosterone leads to increased sodium reabsorption and volume retention as well as potassium secretion, and excessive aldosterone secretion can cause hypertension and hypokalemia^3^.

In 1955, Conn reported a patient with hypertension, muscular weakness, paralysis, polydipsia, polyuria, and hypokalemic alkalosis who was cured after removal of a 4 cm adrenocortical adenoma (benign tumor of the adrenal cortex)^4^. He described this as a new syndrome of “primary aldosteronism” (PA). Currently, PA is recognized as the most common cause of secondary hypertension, accounting for up to 6% of hypertensive patients in primary care and up to 11% of patients in specialized referral centers; its prevalence increases with the severity of hypertension^5,6^. PA is characterized by at least partially autonomous aldosterone production (despite low renin levels), hypertension and low or normal serum potassium levels^7^. Common causes, include idiopathic hyperaldosteronism (IHA; approximately 60% of the cases) and aldosterone-producing adenoma (APA; approximately 30%)^5,6^, and rare subforms include unilateral hyperplasia, adrenocortical carcinoma (ACC), or familial hyperaldosteronism (FH). PA is associated with an increased risk of cardiovascular diseases, such as stroke, myocardial infarction, or atrial fibrillation compared with primary hypertension^8–10^. In patients with hypertension, an elevated aldosterone–renin ratio (ARR) is used as a screening parameter, followed by confirmatory testing (salt loading, fludrocortisone or captopril administration, which all fail to sufficiently lower aldosterone levels in PA). Imaging, such as computed tomography, serves to exclude malignancy, whereas so-called adrenal venous sampling, an invasive procedure, is typically required to distinguish unilateral (mostly APA) from bilateral (usually IHA) aldosterone production^7^. However, adrenal venous sampling remains challenging, and hence, many patients are not diagnosed or treated optimally. While APAs can potentially be cured by surgery, mineralocorticoid receptor antagonists remain the treatment of choice for IHA^7,11,12^.

Despite the well-established role of aldosterone in renal salt handling and blood pressure regulation, the molecular cause(s) underlying PA, with the exception of a rare familial form^13^, were unknown until approximately 8 years ago. In recent years, next-generation sequencing approaches have allowed for the identification of somatic (tumor-specific) mutations in APAs as well as germline mutations in FH (Table 1), offering a completely new perspective on the pathophysiology underlying PA and improving our understanding of the regulation of aldosterone production in the adrenal gland^14–20^. The purpose of this review is to summarize and discuss these findings.

**Table 1:** Genes implicated in elevated aldosterone production in humans and mouse models of PA

*Human*		
Aldosterone-producing adenoma (APA)		
Gene name	Protein name	Common recurrent variants (selection)
* KCNJ5*^16^	G-protein coupled inwardly rectifying potassium channel 4	G151R^16,17,21–23,25,47^ L168R^16,17,21–23,25,47^
* CACNA1D*^14,17^	Ca_V_1.3	G403R^14,17,25,35,62^ I750M^14,17,25,35,62^ F747L^14,25,35^ F747V^17,26,35^ P1336R^14,25^
* ATP1A1*^15^	Na-K-ATPase subunit 1	L104R^15,17,22,25,46,62^ p.F100_L104del^14,15,25^
* ATP2B3*^15^	Plasma membrane calcium ATPase	L425_V426del^15,17,22,25,62^
* CTNNB1*^39^	β-catenin	S45P^17,36^ S45F^98^
Aldosterone-producing cell clusters (APCCs)		
Gene name	Protein name	Variants shared with common APA variants
* CACNA1D*^56^	Ca_V_1.3	G403R, F747L, F747V^56^
* ATP1A1*^56^	Na-K-ATPase subunit 1	L104R^56^
* ATP2B3*^56^	Plasma membrane calcium ATPase	
Idiopathic hyperaldosteronism (IHA)		
* CACNA1D*^59^	Ca_V_1.3	G403R, F747L, F747V^59^
* KCNJ5*^59^	GIRK4	G151R^59^
Unilateral adrenal hyperplasia (UAH)		
* CACNA1D*^37^	Ca_V_1.3	G403R, F747V^37^
Adrenocortical carcinoma		
* KCNJ5*^21^	GIRK4	L168R^21^
Familial hyperaldosteronism		
Gene name	Protein name	Implicated variants
* CYP11B1/CYP11B2* (Type I)^13^	11ß-hydroxylase/aldosterone synthase	Chimeric gene of *CYP11B1* (promotor) and *CYP11B2* (coding region)^13^
* CLCN2* (Type II)^18,19^	ClC-2	R172Q, Y26N, K362del, M22K, S865R^18^, G24D^19^
* KCNJ5* (Type III)^16^	GIRK4	G151R^29^ G151E^29,77^ T158A^16^ E145Q^21^ Y152C^77^
* CACNA1H* (Type IV)^20^	Ca_V_3.2	M1549V^20^ Ser196Leu, p.Pro2083Leu, M1549I^81^
* CACNA1D* (PASNA)^17^	Ca_V_1.3	G403D, I770M^17^
Mouse		
*Mouse model*	Phenotype	
* Kcnj5 KO*^86^	Reduced serum aldosterone	
* Task1 KO*^90^	Expression of *Cyp11b2* in ZF and ZR Severe hyperaldosteronism (hypokalemia, low renin) Glucocorticoid-remediable phenotype	
* Task3 KO*^92,93^	Salt-sensitive hypertension Slightly elevated aldosterone and suppressed renin Resistance to salt suppression	
* Task1* and *Task3 KO*^91^	Depolarization of ZG Hyperaldosteronism Resistance to candesartan and salt suppression	
ZG-specific *Task1* and *Task3 KO*^94^	Mildly elevated aldosterone, suppressed renin Chronic blood pressure elevation	
* Cry1* and *Cry2* KO^96^	Increased aldosterone, suppressed renin Kidney damage No hypertension	
* CYP11B2* coding sequence expression under the control of the *CYP11B1* promoter^88^	Mouse model for FH-I Hyperaldosteronism and elevated BP under HSD Responsive to fadrozole (CYP11B2 inhibitor)	
* SSpo* mice^97^	Upregulation of *Cyp11b2* Elevated ARR	

*ZF* adrenocortical zona fasciculata, *ZR* adrenocortical zona reticularis, *ZG* adrenocortical zona glomerulosa, *BP* blood pressure, *HSD* high-salt diet, *ARR* aldosterone:renin ratio

## Somatic mutations in APA

### Mutations in potassium channel KCNJ5 in APA

In 2011, Choi et al. conducted a whole-exome sequencing study comparing the blood and tumor DNA of four patients with APAs showing hypertension, high ARR, and unilateral adrenal cortical masses upon CT evaluation. The overall number of somatic (tumor-specific) mutations in APAs was low (2.3 protein-altering and 0.8 silent mutations per tumor) in comparison to malignant tumors. In two APAs, Choi et al.^16^ discovered heterozygous somatic mutations in the gene *KCNJ5* (G151R and L168R) encoding the inwardly rectifying potassium channel Kir3.4. Subsequent targeted Sanger sequencing in 18 APAs revealed six additional somatic *KCNJ5* mutations (one G151R and five L168R). These two hotspot mutations were later shown to account for the vast majority of *KCNJ5* mutations in APAs; other mutations are very rare (L168R: 23–44%, G151R: 54–79%, others: 0–4.5%^21–23^). Ensuing studies in large cohorts have revealed that *KCNJ5* mutations may account for approximately 40% of mutations in APAs^21,24,25^, with considerable variation among different ethnicities and among women versus men (see below).

**Fig. 1:**
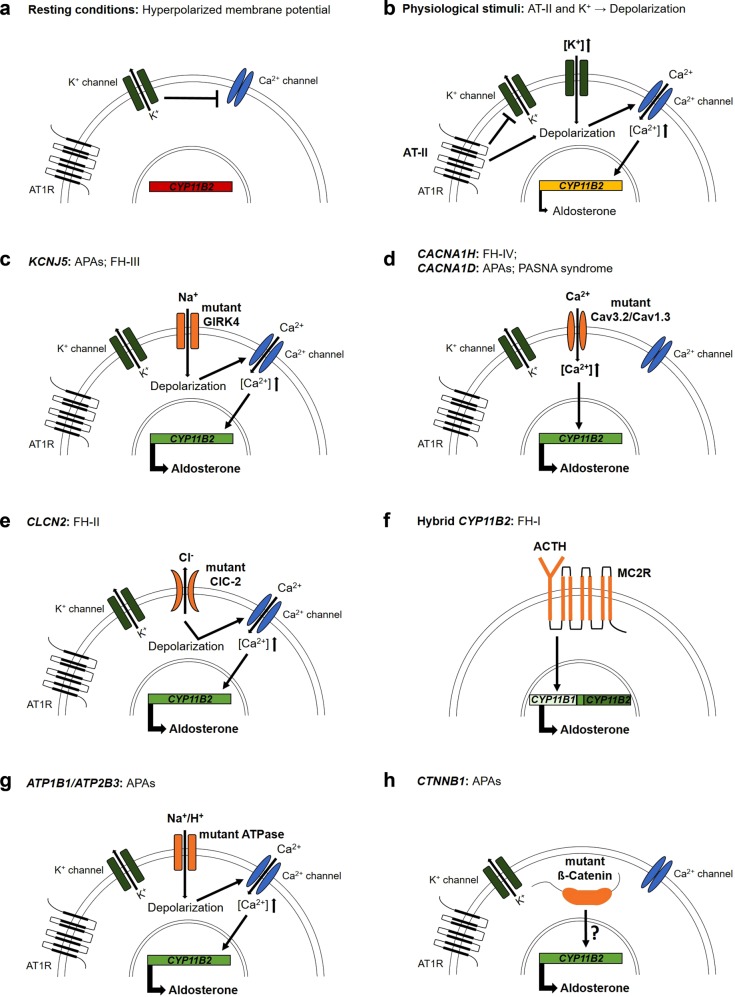
Physiology of adrenal aldosterone production and the mechanism of mutations in PA. **a** Under resting conditions, voltage-gated calcium channels are closed due to a high potassium conductance and the resulting hyperpolarized membrane potential. **b** Physiological stimuli of glomerulosa cells include angiotensin II and hyperkalemia, leading to depolarization, followed by the opening of voltage-gated calcium channels, the signal for aldosterone synthase expression and aldosterone production. **c** *KCNJ5* variants (as somatic mutations in APAs and germline mutations in FH-III) change the ion selectivity of the potassium channel, permitting sodium influx and the depolarization of the cell membrane. **d** Mutations of *CACNA1H* (as germline mutations in FH-IV) and *CACNA1D* (as somatic mutations in APAs and germline mutations in PASNA syndrome) directly cause increased calcium permeability. **e** The higher chloride permeability of *CLCN2* variants (as germline mutations in FH-II) depolarizes glomerulosa cells by chloride efflux. **f** The ACTH-dependent expression of a hybrid variant of *CYP11B2* (as germline mutation in FH-I) in zona fasciculata cells directly increases hybrid gene expression. **g** Variants of *ATP1A1* and *ATP2B3* (as somatic mutations in APAs) lead to increased permeability for sodium or protons and thereby depolarize glomerulosa cells. **h** The underlying mechanism of elevated aldosterone production by variants of *CTNNB1* (as somatic mutations in APAs) is incompletely understood. AT1R, angiotensin II receptor type I; MC2R, adrenocorticotropic hormone receptor

Kir3.4 channels have two transmembrane domains and form tetrameric channels with a central common pore. Potassium selectivity is mostly conferred by the selectivity filter located in the loop between the pore helix and the second transmembrane domain^26^. The selectivity filter contains a signature sequence (TXGYG) shared among numerous different potassium channels^27^. The most common *KCNJ5* mutations in APAs are located either within (G151R) or close to (L168R) the selectivity filter. Both mutations cause abnormal sodium permeability of the mutant channel, which results in a depolarization of the cell membrane (Fig. 1). Based on these findings, Choi et al. proposed that in APAs with *KCNJ5* mutations, tumor formation and autonomous aldosterone production are driven by membrane depolarization of glomerulosa cells, leading to increased calcium influx via voltage-gated calcium channels^1^ and subsequent changes in the expression of genes implicated in proliferation and aldosterone synthesis. Evidence that *KCNJ5* mutations are likely sufficient to cause both aldosterone production and tumor formation is provided by the overall rarity of additional somatic variants in APAs with pathogenic *KCNJ5* mutations, specifically the absence of additional mutations that explain proliferation^16,28^ and the fact that patients with germline *KCNJ5* mutations found in APAs typically develop massive adrenal hyperplasia as well as early-onset, therapy-resistant PA (see below)^16,29^. A proliferative effect of *KCNJ5* mutations is also suggested by the peculiar case of a patient with germline mosaicism in whom adrenal hyperplasia was restricted to those areas of the adrenal gland that carried *KCNJ5* mutations^30^. Despite these considerations, a two-hit model of APA formation has been proposed involving the activation of signaling pathways such as shh (sonic hedgehog signaling molecule) or Wnt/β-catenin leading to abnormal proliferation (first hit) and subsequent acquisition of somatic mutations in driver genes such as *KCNJ5* leading to increased and autonomous aldosterone production (second hit)^31^. In very rare cases, this may be due to germline mutations in tumor suppressor genes (published case with *APC* mutation)^32^.

### Mutations in calcium channel CACNA1D in APA

Two groups independently discovered APAs with somatic mutations in *CACNA1D*, encoding for the α_1_-subunit of an l-type calcium channel (Ca_V_1.3) via exome sequencing^14,17^. The α_1_-subunit consists of four homologous repeats comprising six transmembrane helices (S1–6) each, and calcium selectivity is determined by a pore loop between S5 and S6^33^. Mutations in *CACNA1D* are more scattered throughout the protein compared with *KCNJ5* mutations^14,17^. By electrophysiology, both groups demonstrated that the somatic mutations discovered caused an increase in calcium permeability, which was inferred to directly lead to increased aldosterone production and proliferation (Fig. 1). In addition to *KCNJ5*, *CACNA1D* is the gene with the second highest mutation burden in APAs^14,17,25^, again with variation among different ethnicities (see below). Studies that relied on Sanger sequencing of only a few exons for somatic mutation detection may have systematically underestimated the prevalence of *CACNA1D* mutations (21–42% prevalence in studies using panel sequencing of the entire coding sequence^34,35^ vs. 1.5–10.3% in studies using Sanger sequencing of select regions^25,36,37^).

### Mutations in ATPases ATP1A1 and ATP2B3 in APAs

In 2013, Beuschlein et al. expanded the portfolio of somatic mutations in APAs. They described heterozygous mutations in *ATP1A1* (encoding for a Na^+^–K^+^–ATPase subunit) and *ATP2B3* (encoding for a plasma membrane Ca^2+^ ATPase)^15^. Mutations were clustered within the M4 helix involved in ion binding, suggesting a gain-of-function mechanism; however, the authors originally described the loss of pump function in mutant ATPases (which may contribute to depolarization). Subsequent studies demonstrated that mutations in *ATP1A1*^14^ and *ATP2B3*^38^ cause abnormal Na^+^ or H^+^ permeability (Fig. 1) and enhance aldosterone production in the human adrenocortical H295R cell line, suggesting an underlying pathomechanism similar to that of *KCNJ5* mutations. In comparison to *KCNJ5* and *CACNA1D*, ATPases show a lower mutational burden in APAs, accounting for 3–17% and 1.5–4% of APAs, respectively^15,25,34,35,37^.

### Mutations in CTNNB1 in APAs

In addition to ion channels and ATPases, APAs, in rare cases, also carry gain-of-function mutations in *CTNNB1*, encoding for β-catenin, the effector of the canonical Wnt signaling pathway^17,37,39^. However, the mechanisms underlying *CTNNB1*-mediated APA formation have not been clarified. A universal role of *CTNNB1* mutations in adrenal tumor formation is supported by the fact that mutations in *CTNNB1* are also found in ACC^40^, cortisol-producing adenomas^41,42^ and nonproducing adenomas^39^. Constitutive activation of *CTNNB1* in mice has been demonstrated to trigger adrenal hyperplasia, aldosteronism and, at advanced age, malignancy^43^. In one study, somatic *CTNNB1* mutations were reported in two pregnant women. The authors suggested that adenoma formation and excessive aldosterone production may be driven by pregnancy via enhanced activation of LHCGR (luteinizing hormone/choriogonadotropin receptor)^44^. However, this has been disputed because *CTNNB1* mutations are found in APAs of both male and female (typically nonpregnant) patients^36,45^.

### Distribution of mutations in APAs—frequency, sex, and ethnicity

Recent studies suggest that somatic mutations in APAs may be more frequent than previously thought. Using *CYP11B2* immunohistochemistry for the identification of aldosterone-producing nodules and next-generation sequencing for somatic mutation discovery, Nanba *et al*. demonstrated that up to 87% (males) or 90–91% (females) of tumors carry somatic mutations in one of the genes described above^34,35^. The pathophysiology in the remainder of the cases, however, remains elusive. Further causes may include previously undiscovered, very rare mutations or gene expression changes due to variations in copy number. In fact, a loss of heterozygosity has been reported in a subset of APAs, and such tumors do not carry *KCNJ5* mutations^16^. Mutations in *KCNJ5* appear to be more frequent in females (56–63%; compared to 22–31% in males)^21,24,25^. Higher frequencies in some Asian cohorts (approximately 60–70% of APAs)^46–48^ were in part attributed to different diagnostic criteria; however, a recent study reporting *CACNA1D* as the gene with the highest mutational burden and lower *KCNJ5* mutation frequencies in a cohort of black subjects with APAs suggests that mutation frequencies may indeed be influenced by ethnicity^35^. The reasons underlying sex- and population-specific variation in APA mutation frequencies are currently unknown.

### Implications for diagnosis and therapy

Despite the broad portfolio of somatic mutations and their mechanistic implications discussed above, genetic mutations are currently not exploited for diagnostic or therapeutic purposes. Adrenal venous sampling, the current gold standard for diagnosis, is a complicated and invasive procedure that is available only in specialized centers, impeding high-throughput diagnosis. It has been suggested that selective inhibitors of mutant *KCNJ5* channels have the potential to improve this situation: a drop in blood pressure and/or aldosterone upon administration of such inhibitors could allow for the noninvasive identification of patients with adenomas carrying *KCNJ5* mutations^49^. Because *KCNJ5*-positive tumors are often large, with typical characteristics upon computed tomography^37^, they could possibly be surgically removed without the need for adrenal venous sampling. Interestingly, in a high-throughput screen, macrolide antibiotics such as roxithromycin and clarithromycin were identified as specific inhibitors of both *KCNJ5*^*G151R*^ and *KCNJ5*^*L168R*^, with no significant inhibition of the wild-type channel. Importantly, this effect was also present in macrolide compounds without antibiotic or motilin receptor agonist activity^49^. In a proof-of-principle clinical trial, the antibiotics roxithromycin and clarithromycin (with established safety) are currently being investigated as diagnostic tools^50^. Similarly, genotype-specific steroid profiles have been suggested as diagnostic tools. Specifically, tumors carrying *KCNJ5* mutations are characterized by higher production of the so-called hybrid steroid 18-oxocortisol^51^, which could serve to identify APAs for surgery.

## DNA methylation and APAs

Genome-wide analysis of methylation and corresponding gene expression comparing APA, normal adrenocortical tissue (including the ZG, fasciculata, and reticularis) and nonproducing adenomas demonstrated, among other differentially methylated and expressed genes, higher expression and decreased methylation of *CYP11B2* in APAs^52^, and these results were confirmed in an independent study^53^. How this hypomethylation relates to somatic mutations in APAs is less clear^54^.

## APCC and IHA

Studies using an antibody against aldosterone synthase (*CYP11B2*), one of the rate-limiting factors of aldosterone production, have demonstrated that the human ZG is not always a continuous arrangement of aldosterone-producing cells. Rather, with increasing age, so-called aldosterone-producing cell clusters (APCCs) develop, which express *CYP11B2* and protrude into the zona fasciculata^55–57^. A possible role for APCCs in PA is supported by a study investigating *CYP11B2* expression in adrenal slices from a cohort of 32 patients with PA^58^. Six patients did not show signs of APA despite PA, and 4 of these patients had multiple *CYP11B2*-expressing APCCs. Interestingly, one patient with adenoma showed no expression of *CYP11B2* in the tumor but had multiple *CYP11B2*-expressing APCCs. By subjecting *CYP11B2*-expressing APCCs of normal adrenal glands to panel sequencing of candidate genes, Nishimoto et al. provided evidence that APCCs are genetically similar to APAs^56^. They reported 11 somatic mutations (7 in *CACNA1D*, 3 in *ATP1A1* and 1 in *ATP2B3*) in 31 APCCs, 7 of which were situated at residues affected by known somatic mutations in APAs. This finding also suggests that APCCs may be precursors of APAs. Interestingly, no *KCNJ5* variants were detected. Lesions with *KCNJ5* mutations may quickly progress to APAs and hence may be underrepresented in this study.

In addition to APA formation, APCCs have also recently been implicated in IHA^59^. Omata et al.^59^ identified 15 patients who had undergone unilateral adrenalectomy despite bilateral disease. The resected adrenal glands showed more and larger APCCs than the normotensive controls. Sequencing of 99 APCCs in this cohort revealed *CACNA1D* mutations in 58% and *KCNJ5* mutations in 1% of the lesions. All adrenals had at least one APCC with such a mutation, but the causes of the remaining lesions are currently unknown. Exome sequencing of DNA extracted from APCCs will likely provide additional insight. To confirm the role of APCCs in IHA, it would be interesting to study the adrenal glands of subjects who continue to have elevated aldosterone levels after adrenalectomy (as a potential sign of bilateral disease).

Building on these observations, one could speculate that APCCs may also contribute to subclinical PA in a subset of patients who suffer from hypertension but do not meet the criteria for the diagnosis of PA (see above). If proven to be true, this assumption may help explain findings from the PATHWAY-2 study demonstrating that the mineralocorticoid receptor antagonist spironolactone is the most effective add-on drug in treatment-resistant hypertension^60^. However, the use of thiazide or thiazide-like diuretics and the resulting upregulation of the renin–angiotensin–aldosterone axis may also lead to increased spironolactone sensitivity.

## Unilateral adrenal hyperplasia and PA

Together, bilateral adrenal hyperplasia and APA account for more than 90% of patients with PA. However, a small subset of patients with PA (~2%) may also present with diffuse unilateral adrenal hyperplasia (UAH)^61^, although such cases should be studied in more detail using *CYP11B2* immunohistochemistry. Unlike for APAs, the genetic causes underlying diffuse UAH are largely unknown. One study reported *CACNA1D* mutations in two patients with PA and UAH^37^, suggesting a common underlying pathomechanism in UAH and APAs. In contrast, nodular UAH is fairly common. In many cases, one nodule expresses aldosterone synthase and carries a known APA driver mutation, whereas other nodules represent nonproducing adenomas or nodules without such mutations, but two APAs in a single gland with independent driver mutations have also been reported^62^.

## Genome-wide association studies

Spyroglou et al.^63^ performed a genome-wide association study of probands from the German KORA (Cooperative Health Research in the Augsburg Region) cohort and reported a significant association of the aldosterone:renin ratio (but not PA) with a locus on chromosome 5q32 comprising the *SLC26A2* gene. Based on cell and mouse studies, they suggested a functional role of SLC26A2 in aldosterone production. However, to our knowledge, this association has not been independently replicated. Other groups have performed candidate gene association studies^64,65^.

## Adrenocortical carcinoma

ACC is a very rare disease in the general population (incidence of up to 2 cases per million per year) with a dismal prognosis (5-year overall survival 37–47%)^66^. Clinically, a large proportion of patients present with overproduction of adrenal steroid hormones, most commonly hypercortisolism^66^. In rare cases, ACC patients may also present with hyperaldosteronism, featuring increased plasma aldosterone despite low plasma renin activity, hypertension and hypokalemia^67,68^, and one case of aldosterone-producing ACC carrying the *KCNJ5*^*L168R*^ mutation has been reported^21^.

## Familial hyperaldosteronism

FH is a rare cause of PA. Thus far, heterozygous variants in the underlying disease genes have been identified in five subtypes, which are all inherited in an autosomal-dominant fashion or occur de novo (see below). The proportion of patients with unexplained FH is difficult to assess, but descriptions of unsolved cases with early-onset PA suggest that additional genes remain to be discovered^18^.

### Familial hyperaldosteronism type I

In 1966, Sutherland et al.^69^ described a father and his son who presented with hypertension, increased aldosterone levels despite suppressed plasma renin activity and potassium deficiency. Surprisingly, all abnormalities were relieved by dexamethasone administration^69^; hence, the syndrome was named glucocorticoid-remediable aldosteronism (GRA). After the discovery of additional forms of FH, it was also referred to as FH-I. Many years after the initial description of the syndrome, the molecular basis of the response to glucocorticoids was identified. As a result of unequal crossing over between the highly homologous genes 11ß-hydroxylase (*CYP11B1*) and aldosterone synthase (*CYP11B2*), a chimeric gene is formed. This chimeric gene combines the adrenocorticotrophic hormone (ACTH)-responsive regulatory sequences of *CYP11B1* with the coding sequence of *CYP11B2*, leading to ectopic expression of *CYP11B2* in the zona fasciculata under the control of ACTH^13,70^. The chimeric gene was detected in 29 patients from 12 unrelated pedigrees, including the two patients described by Sutherland et al.^13,69^. Not all subjects with the chimeric gene are hypertensive, but even in those who are normotensive, excessive aldosterone levels lead to increased left ventricular wall thickness and reduced diastolic function compared to normotensive controls^71^. The diagnosis is based on genetic testing; therapeutic options include mineralocorticoid receptor antagonists and glucocorticoids^7^.

### Familial hyperaldosteronism type II

The term FH-II was initially coined for familial aggregation of PA without response to glucocorticoid administration^72^. Later, a diagnosis of FH-II was made when at least two first-degree members (offspring, sibling, and parent) of the same family had confirmed PA^73^; this included both patients with IHA and APA. However, because sporadic PA is not a rare disorder, the majority of such cases are likely chance associations and do not have Mendelian disorders^74^. Of the published kindreds, one already described in the early 1990s stood out because of the large number of affected subjects^72^, but linkage analysis failed to identify the underlying disease gene^75^. Exome sequencing of selected individuals from this kindred demonstrated a mutation in the *CLCN2* gene, encoding the chloride channel ClC-2. An investigation of 80 individuals with early-onset unexplained PA revealed seven additional subjects with *CLCN2* mutations, and it was suggested to refer to individuals with *CLCN2* mutations as FH-II^18^. In a parallel publication, one further case was identified^19^. FH-II subjects typically had elevated ARR (although incomplete penetrance was observed) and, where studied, nonlateralizing aldosterone production; massive adrenal hyperplasia was absent^18,19^.

*CLCN2* mutations cause a gain of function. Increased chloride permeability in the presence of high intracellular chloride concentrations leads to depolarization and voltage-gated calcium influx, followed by *CYP11B2* upregulation^18,19^.

### Familial hyperaldosteronism type III

In 2008, Geller et al. described a family (a father and his two daughters) with a new form of PA that did not respond to dexamethasone. The affected subjects showed severe early-onset aldosteronism, including the production of hybrid steroids and massive adrenocortical hyperplasia^76^. Because medical treatments failed, all three subjects underwent bilateral adrenalectomy^76^. In conjunction with the identification of somatic *KCNJ5* mutations in APAs, a germline *KCNJ5* mutation (T158A) was found in this family;^16^ this mutation is also rarely found in APAs^77^. Since then, other families with mutations in or close to the *KCNJ5* selectivity filter have been reported.

With some exceptions, *KCNJ5* mutations that are also found in APAs, when present in the germline, appear to be associated with the development of macroscopic adrenal hyperplasia and a severe phenotype. Scholl et al. identified a germline G151R mutation (most common somatic mutation in APAs (see above)) in three individuals of one kindred who all required bilateral adrenalectomy and a de novo variant in a fourth unrelated patient^29^. Similarly, E145Q (found in an APA^21^) was reported as a de novo mutation in a female patient diagnosed with PA at 2 years of age due to massively elevated aldosterone levels and blood pressure, treatable only by bilateral adrenalectomy^78^.

In contrast, mutations that are only found in the germline are typically associated with a milder clinical phenotype without macroscopic adrenal hyperplasia and without requirement for bilateral adrenalectomy. Such mutations include the G151E mutation found in three kindreds who responded to treatment with mineralocorticoid antagonists^29,77^. Similarly, a Y152C mutation was found in a patient diagnosed at the age of 48 years with hypertension and a milder phenotype^79^. Functional studies suggest that while all variants affect the ion selectivity of the channel, G151E causes more profound sodium permeability, which may impair cell survival and prevent the development of hyperplasia^29^. These findings again support a role for APA-associated *KCNJ5* mutations not only in aldosterone production but also in cellular proliferation (see above). The term “familial hyperaldosteronism type III” (FH-III) was initially used to describe a phenotype of dexamethasone-refractory hyperaldosteronism with massive adrenal hyperplasia. However, following the description of patients with *KCNJ5* mutations with variable phenotypes, FH-III has been used to refer to PA patients with germline *KCNJ5* mutations.

### Familial hyperaldosteronism type IV

To identify new disease-causing genes in FH, Scholl et al.^20^ examined 40 unrelated individuals diagnosed with PA until the age of 10 years by exome sequencing. Five unrelated subjects shared the identical novel heterozygous mutation of the voltage-gated T-type calcium channel CACNA1H (M1549V). Family analysis demonstrated the de novo occurrence of the mutation in two kindreds and also suggested incomplete penetrance^20^. The mutant calcium channel showed dramatically impaired channel inactivation and activation at more hyperpolarized membrane potentials, resulting in increased intracellular Ca^2+^, the signal for aldosterone production^20^. The expression of the mutant channel in H295R cells and its subclone HAC15 resulted in increased aldosterone production compared to the wild-type channel, which was abolished by the inhibition of CACNA1H channels with the T-type calcium channel blocker mibefradil^80^. Further *CACNA1H* germline variants were identified by whole-exome sequencing in patients with different types of PA: another de novo case carrying M1549I in a patient with early-onset PA; S196L in a brother and a sister; P2083L in brothers diagnosed with FH; and V1951E in a patient with APA^81^. Whether variants at residues other than M1549 are causative for FH-IV, however, remains to be established.

### PA with seizures and neurological abnormalities (PASNA syndrome)

In addition to somatic mutations in APAs, the *CACNA1D* gene also shows germline variants in PA. Due to the associated very severe phenotype, such variants exclusively occur de novo.

Scholl et al. investigated 100 unrelated individuals with unexplained early-onset PA and identified de novo *CACNA1D* germline mutations in two subjects: G403D in a patient with hypertension at birth along with cardiac pathophysiology (e.g., ventricular septal defect) and neurological disorders (e.g., seizures and apparent cerebral palsy), and I770M in a subject with cerebral palsy and seizures since birth and elevated blood pressure at 5 years of age^17^. By electrophysiology, mutant channels showed activation at less depolarized membrane potentials and impaired inactivation in G403D mutants, which were inferred to cause increased Ca^2+^ influx and *CYP11B2* upregulation^17^. A de novo G403D mutation was also identified in a subject with persistent congenital hyperinsulinaemic hypoglycemia (HH), mild aortic insufficiency, and seizures^82^. Aldosterone was within the normal range, but renin was not determined. Finally, de novo *CACNA1D* variants with similar functional consequences as in PA have been implicated in autism and epilepsy^83–85^. These observations likely represent different manifestations of a complicated syndrome.

## Genetic mouse models

While genetic mouse models replicating human FH mutations are sparse, PA mouse models with mutations in genes hitherto not implicated in FH have been generated, in part due to species differences in the adrenal expression of ion channels.

Perhaps surprisingly, given the low expression levels of *Kcnj5* in rodents, a *Kcnj5* knockout model showed reduced aldosterone levels in female but not in male mice^86^. A model with adrenal expression of wild-type and mutant human *KCNJ5* under the control of the *Akr1b7* promoter has been reported only in abstract form but did not show adrenal hyperplasia or severe aldosteronism^87^. Another transgenic mouse model expresses human *CYP11B2* under the control of a human *CYP11B1* promoter mimicking the condition of FH-I^88^. Transgenic mice showed increased aldosterone levels and elevated blood pressure induced by a high-salt diet (4% NaCl), which were significantly reduced by treatment with fadrozole (an inhibitor of *CYP11B2*)^88^.

Because *Kcnj5* is expressed only at low levels in the adrenal gland of rodents, with TWIK-related acid-sensitive potassium (Task) channels as the predominant K^+^ conductance^89^, several TASK mouse models have been created and described. *Kcnk3* (TASK-1) knockout mice show the expression of *Cyp11b2* in the reticulo-fasciculata zone instead of in the ZG and develop severe hyperaldosteronism (including hypokalemia and low renin levels) remediable by glucocorticoids, representing clinical features of FH-I^90^. The disruption of both *Kcnk3* and *Kcnk9* (TASK-3) in mice leads to a depolarization of adrenal glomerulosa cells and an overproduction of aldosterone resistant to both salt suppression and treatment with candesartan along with unchanged or lower renin concentrations^91^. Deletion of only *Kcnk9* leads to salt-sensitive hypertension, slightly elevated aldosterone levels and low plasma renin levels^92,93^. Zona-glomerulosa-specific disruption of *Kcnk3* and *Kcnk9* causes mild autonomous aldosterone production despite low renin and thereby chronic blood pressure elevation^94^. In humans, *KCNK3* (TASK-1) single-nucleotide polymorphisms have been associated with blood pressure traits and aldosterone levels^95^.

To investigate the pathological effects of aldosterone on kidney function, a cryptochrome-null mouse (deletion of cryptochrome genes 1 and 2, which are involved in circadian rhythm) was created^96^. Mice had elevated aldosterone levels and low plasma renin, and kidney injury was present in mice on a normal-salt diet. However, hypertension, as a key feature of PA, was missing^96^.

Last, by treating a cohort of mice with the mutagen N-ethyl-N-nitrosourea (ENU), a mouse line with elevated aldosterone levels was generated^97^. These mice were carriers of point mutations in seven candidate genes, of which a variant in SCO-spondin (*SSpo*) was suggested as the most promising based on a phenotype of increased aldosterone values, associated *Cyp11b2* upregulation as well as elevated ARR^97^.

## Discussion

Taken together, the insights gained over the past decade, mainly through next-generation sequencing, suggest that PA is largely a genetic disease caused by somatic and, in rare cases, germline mutations. Sporadic PA is likely a disease continuum that starts with a somatic driver mutation in a single adrenal cell and subsequently progresses to APCC and/or APA. Recent studies suggest that similar pathophysiology applies to IHA. If this is true, cutoff values for the definition of PA^7^ are, to some degree, arbitrary, and it may be worth retesting patients with borderline results after a few years, in particular if hypertension is difficult to control. Similarly, lesions may initially be too small to be detected by routine imaging. Recent descriptions of APCCs also suggest that the distinction between unilateral and bilateral disease may not be as clear-cut as previously thought, e.g., a patient could have a predominant adenoma on one side and APCCs on the contralateral side.

The partially overlapping mutational spectrum between APAs, APCCs, and FH is also interesting. *KCNJ5* variants explain a substantial portion (approximately 40%) of the somatic mutations in APAs but are also the basis of FH-III. *CACNA1D* variants are involved as somatic mutations in APAs as well as in an inherited form of PA, PASNA syndrome. So far, *CACNA1H* mutations have been reported only as germline mutations in FH-IV, similar to *CLCN2* mutations in FH-II and *CYP11B2* mutations in FH-I; whether they account for rare instances of APAs remains to be determined. In addition to ion channels, somatic mutations in ATPases *ATP1A1* and *ATP2B3* have been implicated in APA formation. Such mutations have not been reported in FH, likely because they would be lethal at the germline level.

Whereas a role of voltage-gated ion channels in adrenal aldosterone production had been well established prior to these studies^1^, *KCNJ5* had not been identified as a key player in aldosterone production, likely due to expression differences between rodents and humans^89^. Similarly, the study of FH-II first pointed to an anion channel in connection with PA and hypertension.

The underlying pathophysiology of the vast majority of mutations appears to involve increased calcium influx, followed by upregulated *CYP11B2* expression and aldosterone production. This increased calcium influx can occur directly, via mutations in voltage-gated calcium channels, or indirectly, via changes in ion permeability or selectivity that cause the depolarization of glomerulosa cells and the subsequent opening of voltage-gated calcium channels. The latter mechanism applies to *KCNJ5*, *CLCN2*, and ATPase mutations. These results suggest that calcium signaling could be a useful therapeutic target in PA. However, approved calcium channel inhibitors are not efficient inhibitors of CACNA1D or CACNA1H channels, and widespread extra-adrenal expression could lead to side effects. Distinct from all other mutations is the formation of a hybrid gene by unequal crossing over between *CYP11B2* and *CYP11B1* along with an aberrant expression pattern of *CYP11B2* in the adrenal cortex, leading to ACTH-dependent aldosterone overproduction in FH-I. The exact pathophysiology of aldosteronism due to *CTNNB1* mutations is less understood.

Recent studies with improved sensitivity suggest that the vast majority of APAs are explained by somatic mutations in either of the genes discussed above. Mutations in additional genes may account for small percentages of APAs. In contrast, many cases of APCCs and early-onset PA remain unexplained, suggesting that additional mutations remain to be identified.

Beyond the mouse models discussed in this review, future animal models replicating the gain-of-function mechanisms implicated in human FH would be interesting tools to study the physiology and pathophysiology of these genes in the adrenal gland. The translation of the genetic findings from the last decade into the clinic is eagerly awaited.
